# Characterizing circulating nucleosomes in the plasma of dogs with hemangiosarcoma

**DOI:** 10.1186/s12917-021-02934-6

**Published:** 2021-06-29

**Authors:** Heather Wilson-Robles, Tasha Miller, Jill Jarvis, Jason Terrell, Theresa Kathleen Kelly, Thomas Bygott, Mhammed Bougoussa

**Affiliations:** 1 College of Veterinary Medicine, Small Animal Clinical Sciences Department, Texas A&M University, College Station, TX 77843 USA; 2 Volition America & Volition Veterinary Diagnostic Development, 13215 Bee Cave Parkway, Galleria Oaks B, Suite 125, Austin, Texas 78738 USA; 3 Volition Diagnostics UK Ltd, 93-95 Gloucester Place, London, W1U 6JQ UK; 4 Belgian Volition SRL, 22 Rue Phocas Lejeune, Parc Scientifique Crealys, 5032 Isnes, Belgium

**Keywords:** Hemangiosarcoma, Dog, Canine, Nucleosome, H3.1, Cell free DNA, Plasma, H3

## Abstract

**Background:**

Nucleosomes consist of DNA wrapped around a histone octamer core like thread on a spool to condense DNA as chromatin into chromosomes. Diseases such as cancer or inflammation lead to cell death, chromatin fragmentation and release of nucleosomes into the blood. The Nu.Q™ platform measures circulating nucleosomes in the blood of humans that result from disease and has been used to detect and identify cancer even at early stages. The objectives of this study are to quantify and better characterize nucleosomes in dogs with various stages of hemangiosarcoma (HSA) using this ELISA-based assay.

Samples from 77 dogs with a confirmed diagnosis of hemangiosarcoma and 134 healthy controls were utilized for this study. The HSA samples were recruited from the Texas A&M University Small Animal Clinic (TAMU-SAC) or purchased from biobanks. All control samples were recruited from the TAMU-SAC.

**Results:**

Dogs with hemangiosarcoma had a 6.6-fold increase in their median plasma nucleosome concentrations compared to controls (AUC 92.9 %). Elevated nucleosome concentrations were seen at all stages of disease and nucleosome concentrations increased with the stage of the disease.

**Conclusions:**

Plasma nucleosome concentrations are a reliable way to differentiate dogs with hemangiosarcoma from healthy dogs. Further testing is underway to better characterize cancer associated HSA circulating nucleosomes and optimize future diagnostics for canine HSA detection.

**Supplementary Information:**

The online version contains supplementary material available at 10.1186/s12917-021-02934-6.

## Introduction

Liquid biopsy (LB) techniques refer to the minimally invasive approach of sampling tumor specific biomarkers in the blood of diseased patients [1]. LB has been most studied in the field of oncology; however, literature also exists on the use of LB for the prognostication of inflammation, sepsis and trauma in humans and dogs [2–5]. In general, LB techniques are used to identify intact circulating tumor cells, extracellular nucleic acids such as cell free DNA (cfDNA), messenger RNA (mRNA), microRNA (miRNA), extracellular vesicles (exosomes), nucleosomes and glycoproteins or antigens in the circulation (e.g. PSA, CEA, CA125) [1].

The detection of cfDNA in the blood of cancer patients is a rapidly emerging field in cancer diagnostics [6]. These techniques allow for the minimally invasive detection of cancer DNA as an adjunct or even as an alternative to standard biopsy approaches. cfDNA can be found in the cell free component of the blood and is typically in fragments that are 120–200 bp long [6]. This cfDNA is often released into the bloodstream through apoptosis or necrosis, therefore, healthy individuals typically have low levels of cfDNA in their plasma [4, 7]. Plasma concentrations of cfDNA are also very dynamic as these fragments are often cleared from the plasma within an hour [4, 7]. Many conditions can influence plasma concentrations of cfDNA including cancer, heavy exercise, inflammation, surgery or tissue injury [8].

Nucleosomes are a particular form of cfDNA released during apoptosis or necrosis in a variety of diseases. They are small fragments of chromosomes that are composed of a 147 bp segment of DNA wrapped around a histone core made of four histones present in duplicate forming an octamer [9]. Nucleosomes have been used as epigenetic biomarkers for the detection or monitoring of a variety of cancers in humans including pancreatic, lung, and colorectal cancer [10–13]. Letendre et al. published some of the first work describing the use of nucleosomes in dogs with trauma and sepsis in 2018 [14, 15]. Recently, our group published data demonstrating elevated nucleosome concentrations in a small cohort of dogs with lymphoma [16]. However, the nucleosome compartment is a relatively uninvestigated area of circulating tumor biomarkers in dogs.

The purpose of this study was to determine if dogs with hemangiosarcoma (HSA), an aggressive mesenchymal tumor derived from malignant endothelial cell precursors, demonstrated elevated concentrations of plasma nucleosomes when compared to healthy dogs [17]. HSA is a devastating disease of dogs and represents 12–21 % of the mesenchymal tumors in this species [18, 19]. This cancer is characterized by rapid progression and early metastasis with a one-year survival rate of < 10 % [20–23]. The most common primary tumor site is the spleen with HSA representing 45–51 % of all splenic malignancies in dogs [20, 21]. The most common presenting complaint for this disease is acute hypovolemic shock due to a spontaneous bleeding episode, however, splenic masses may be found incidentally in many older dogs. For these reasons, HSA is a prime candidate for the development of non-invasive liquid biopsy diagnostics as it would enable a non-invasive cancer screening method that would prevent unnecessary exploratory surgeries which have associated morbidities.

## Results

### Canine patient characteristics

A total of 134 healthy dogs were recruited for this study ranging in age from 10 months to 14 years (median 6 years). Forty-eight of the 134 dogs were aged 7 years or older (35.8 %). The healthy control cohort had a male to female ratio of 1.05 and a sex distribution including 4 intact females, spayed females (*n* = 61), intact males (*n* = 3), and castrated males (*n* = 65). The mean body weight for all healthy dogs was 23 kg (range 2.5 to 55.8 kg). The majority of these dogs were mixed breed dogs (29/134, 21.6 %). Other common breeds included Labrador retriever (*n* = 15), Australian cattle dog (*n* = 10), pit bull terrier (*n* = 7), border collie (n = 6), golden retriever (*n* = 5), dachshund (*n* = 4), and German shepherd (*n* = 3).

There was a total of 77 dogs in the HSA cohort. These dogs ranged in age from 4 to 15 years (median age 10 years). There were 33 spayed females (42.9 %), 2 intact females (2.6 %), 33 neutered males (42.9 %) and 9 intact males (11.7 %) in this cohort. The majority of these dogs were also mixed breed dogs (23/77, 29.9 %). Other common breeds in this cohort included golden retrievers (10/77, 13 %), German Shepherd Dogs (5/77, 6.5 %), Labrador retrievers (4/77, 5.2 %) and 3 Boxers (3.9 %). The remaining dog breeds contained two or fewer patients or were undefined. As expected, the majority of the dogs in this cohort presented with primary splenic HSA (53/77, 68.8 %). Other common sites included the right atrium (7/77, 9.1 %) and bone (5/77, 6.5 %), primary renal (5/77, 6.5 %), subcutaneous/cutaneous (5/77, 6.5 %) and 1 each primary hepatic and skin tumors (1/77, 1.3 %). HSA was confirmed via histopathology in all 77 cases. Tumor stage was available for 64 of the dogs in this cohort. There were 9 dogs with stage I disease (13.8 %), 25 of the cases were considered stage II (38.5 %) and 30 were considered stage III (40.0 %) at diagnosis.

### Nucleosome concentrations are elevated in dogs with HSA

**Fig. 1:**
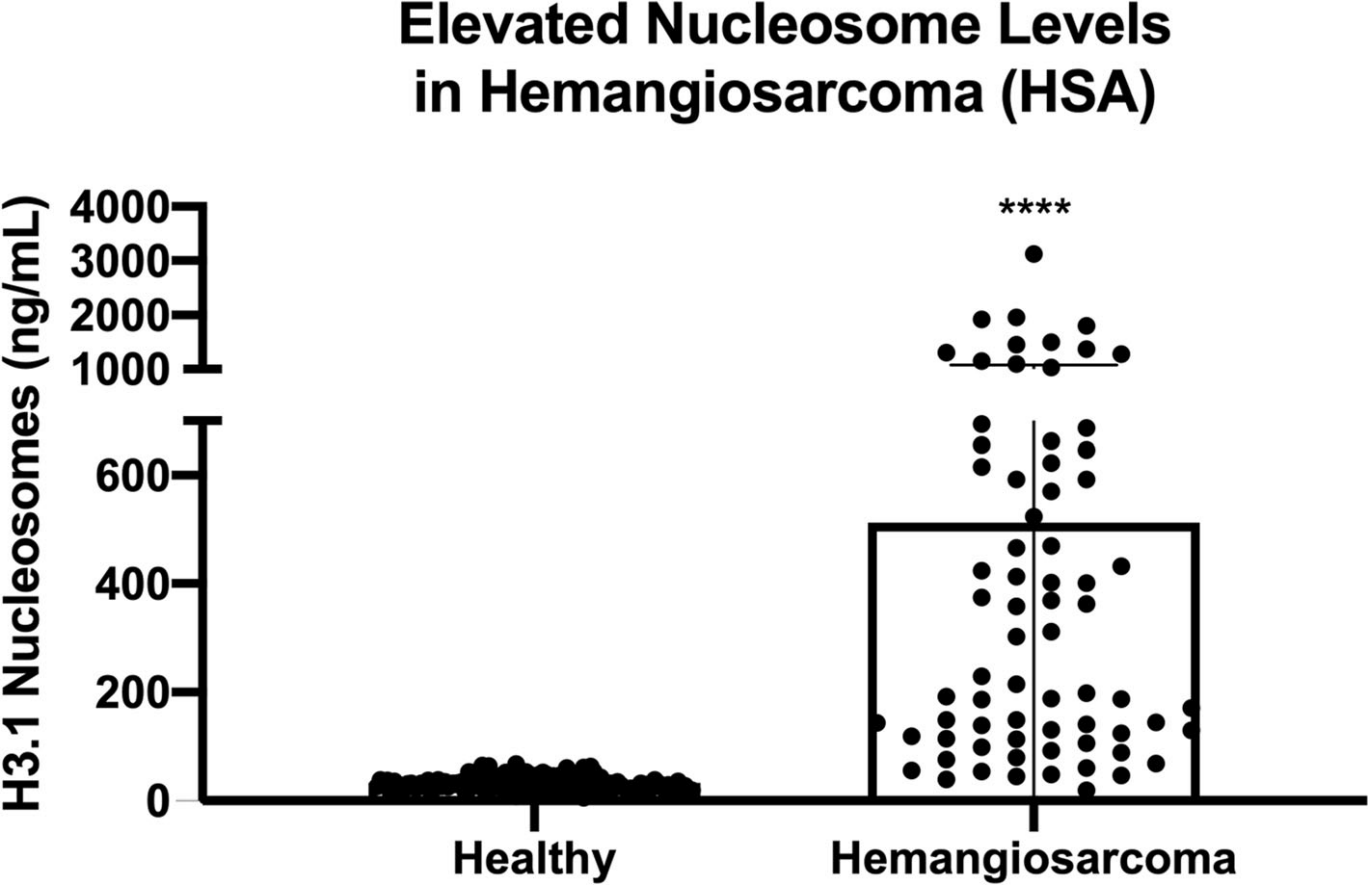
Mean nucleosome concentrations in the plasma of healthy dogs and dogs with HSA

**Fig. 2:**
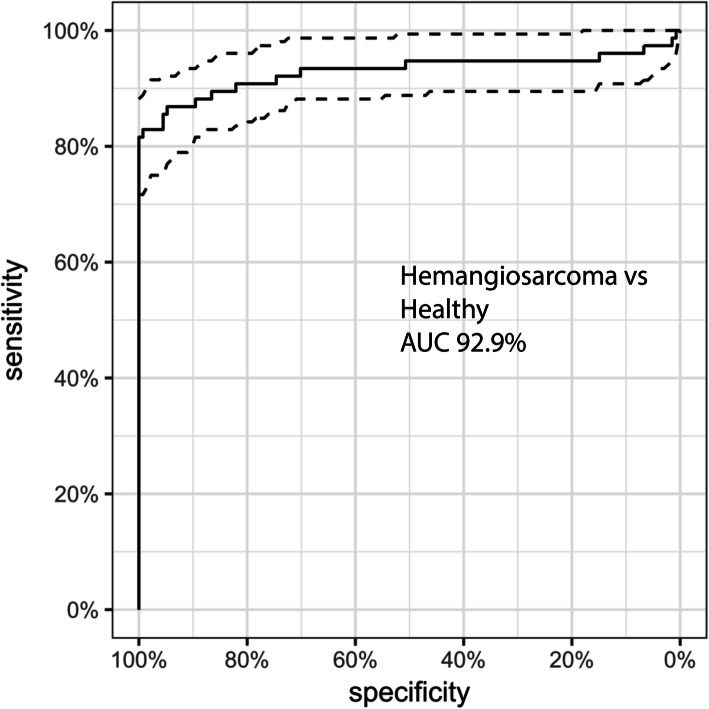
ROC curve demonstrating the ability of the assay to discriminate between healthy dogs and those with HSA. The AUC for this assay was determined to be 92.9 %

Total H3.1 nucleosome concentrations were measured in duplicate for all cases. The median nucleosome concentration for the healthy dogs was 31.1 ng/mL (mean 32.1 ng/mL, SEM 1.1). The dogs with confirmed HSA had a 6.6-fold increase in the median nucleosome concentration at 204.6 ng/mL (mean 420.0 ng/mL, SEM 53.6, *p* < 0.0001) when compared to the healthy dogs (Fig. 1). A receiver operator characteristic (ROC) curve was generated using these data and the area under the curve (AUC) was calculated to be 92.9 %. For a specificity of 100 % the cut off for the healthy range was set at 67.5 ng/mL (nucleosome range for all healthy dogs was 6.3–67.4 ng/mL). At this concentration, the sensitivity of this assay was 80.5 % and the specificity was 100 % (Fig. 2).

### Elevated nucleosome concentrations vary by location in dogs with HSA

**Fig. 3:**
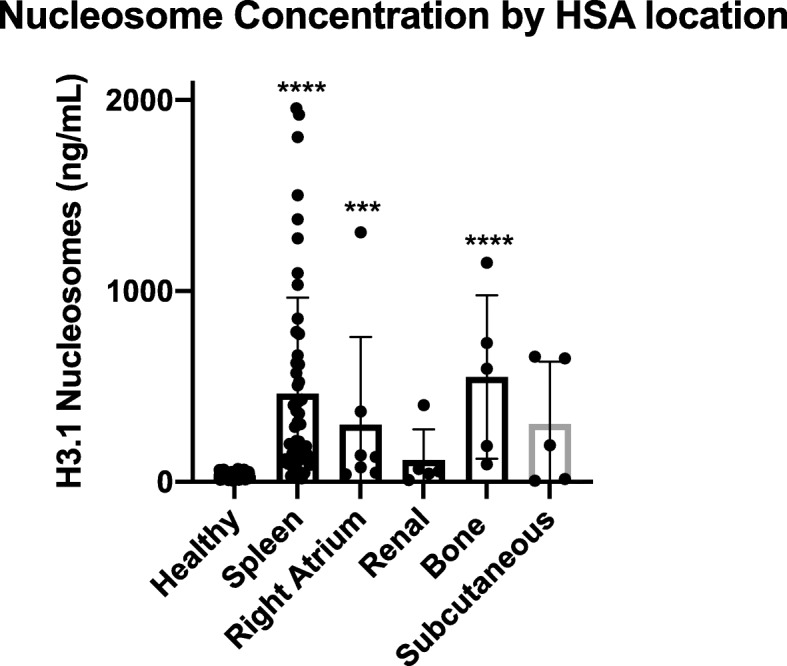
Median nucleosome concentrations in dogs with HSA based on primary location. Dogs with primary splenic, cardiac and bone HSA had significantly higher median nucleosome concentrations than healthy dogs

Plasma nucleosome concentrations were evaluated for those locations with at least five cases each (Fig. 3). The median nucleosome concentration for dogs with primary splenic HSA regardless of stage was 214.8 ng/mL (*n* = 53, mean 461.6 ng/mL, SEM 69.4, *p* < 0.0001). For dogs with primary cardiac HSA the median nucleosome concentration was 129.9 ng/mL (*n* = 7, mean 300.9 ng/mL, SEM 172.9, *p* = 0.0001. For those with primary osseous HSA the median nucleosome concentration was 592.8 ng/mL (*n* = 5, mean 549.8 ng/mL, SEM 191.5, *p* = 0.00015. Though the median nucleosome concentration for dogs with subcutaneous HSA was high, this group was not significantly different from the healthy cohort (*n* = 5, median 331.0 ng/mL, mean 331.0 ng/mL, SEM 184.8, *p* = 0.904). The group with the lowest median nucleosome concentration were those with primary renal HSA (*n*-=5, median 55.5 ng/mL, mean 115 ng/mL, SEM 72.2, *p* = 0.068. With the designated cut off of 67.5 ng/mL the test was able to positively predict HSA in 47/53 (88.7 %) cases of splenic HSA, 5/7 (71.4 %) primary cardiac HSA cases, 5/5 (100 %) primary bone HSA cases, 3/5 (60 %) primary SQ HSA cases and 1/5 (20 %) primary renal HSA cases.

### Elevated nucleosome concentrations are seen in all three stages of HSA

**Fig. 4:**
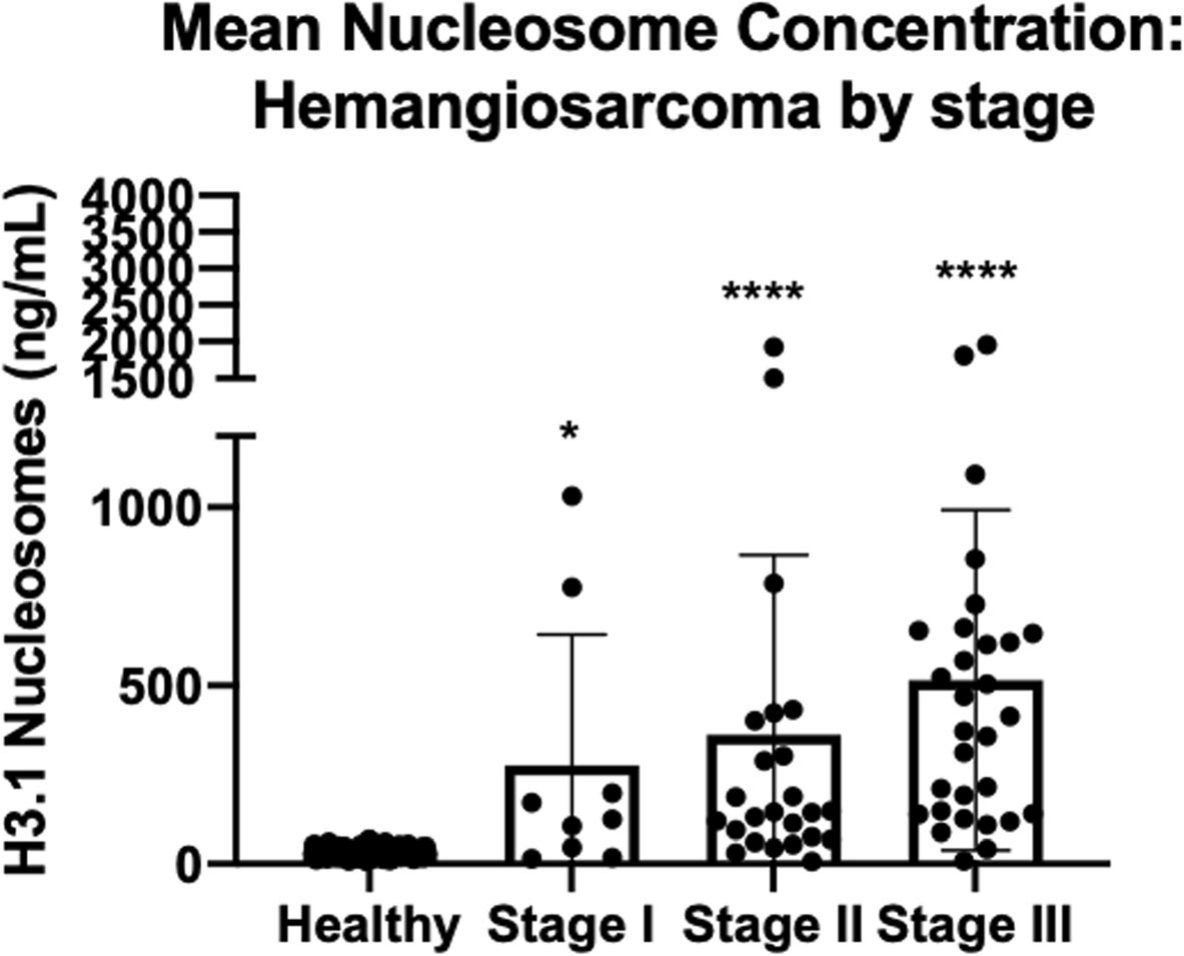
Mean Nucleosome concentrations from dogs of all stages with HSA compared to healthy controls. The assay was able to detect 67 % of dogs with stage I, 76 % of dogs with stage II and 90 % of dogs with stage III disease

The 64 dogs with HSA for whom staging information was available were assessed to determine if nucleosome concentrations could be used to identify those with early stage disease. Using the previously determined healthy range cut off of 67.5 ng/mL, the assay was able to detect 6 of 9 cases with stage I disease (66.7 %), 19/25 cases with stage II disease (76 %) and 27 of 30 cases with stage III disease (90 %). Interestingly, the 2 stage III dogs not detected by the assay both had primary renal HSA. Median nucleosome concentrations increased with the stage of disease. The median nucleosome concentration for dogs with stage I disease was 124.2 ng/mL (mean 276.3 ng/mL, SEM 122.3, *p* = 0.04, AUC 78.7 %). The median nucleosome concentration for dogs with stage II disease was 144 ng/mL (mean 361.6 ng/mL, SEM 100.8, *p* < 0.0001, AUC 92.9 %) and the median plasma concentration for dogs with stage III disease was 413.1 ng/mL (mean 521.9 ng/mL, SEM 89.4, *p* < 0.0001, AUC 95.7 %) (Fig. 4).

### Nucleosome concentrations in dogs with HSA are not affected by age, sex or weight

All 77 dogs had a reported age and gender. Sixty-nine of the dogs had a reported body weight (kg). Dogs with HSA were separated into 3 groups based on age (4–7 years *n* = 18, 8–11 years *n* = 39 and 12–15 years *n* = 20). All three age groups had significantly higher mean and median nucleosome concentrations than healthy dogs (Table 1). Dogs with HSA were separated into 4 groups based on gender (female spayed *n* = 33, female intact *n* = 2, neutered male *n* = 33, and male intact *n* = 9). There were too few cases in the female intact group for a meaningful comparison, however there were enough cases in the other groups for comparisons to be made (Table 2). Finally, dogs with HSA were separated into 4 groups based on weight. The 4 weight groups were < 15 kg *n* = 5, 15–30 kg *n* = 25, 30–45 kg *n* = 34, and > 45 kg *n* = 5. All groups had significantly higher median nucleosome concentrations except for the dogs > 45 kg (*p* = 0.057) than the control dogs, however, there were very few dogs in the < 15 kg and > 45 kg groups (Table 3). When compared to each other, there was no significant difference between the groups of HSA cases in any category (*p* > 0.999 for all comparisons).

**Table 1:** Nucleosome concentrations in dogs with hemangiosarcoma separated by age

	Control	4–7 years	8–11 years	12–15 years
Number of cases	134	18	39	20
Median	31.1	140.7	210.9	214.1
Mean	32.1	366.2	408.1	488.7
SEM	1.1	94.8	74.3	122.1
*P* value (healthy comparison)		< 0.0001	< 0.0001	< 0.0001
*P* value (compared to other HSA groups)		> 0.999	> 0.999	> 0.999

**Table 2:** Nucleosome concentrations in dogs with hemangiosarcoma separated by gender

	Control	Female Spayed	Female Intact	Male Neutered	Male Intact
Number of cases	134	33	2	33	9
Median	31.1	258.8	185.3	187.5	148.6
Mean	32.1	408.1	185.3	473.4	318.4
SEM	1.1	74.3	44.6	96.7	101.4
*P* value (Healthy comparison)		< 0.0001	0.0158	< 0.0001	< 0.0001
*P* value (compared to other HSA groups)		> 0.999	> 0.999	> 0.999	> 0.999

**Table 3:** Nucleosome concentrations in dogs with hemangiosarcoma separated by weight

	Control	up to 15 kg	above 15 up to 30 kg	above 30 up to 45 kg	above 45 kg
Number of cases	134	5	25	34	5
Median	31.1	129.9	132.1	212.9	113.7
Mean	32.1	462.2	309.3	422.5	195.3
SEM	1.1	336.2	61.8	79.7	74.6
*P* value (healthy comparison)		0.00015	< 0.0001	< 0.0001	0.0126
*P* value (compared to other HSS groups)		> 0.999	> 0.999	> 0.999	> 0.999

### Nucleosome concentrations are altered with response to therapy

**Fig. 5:**
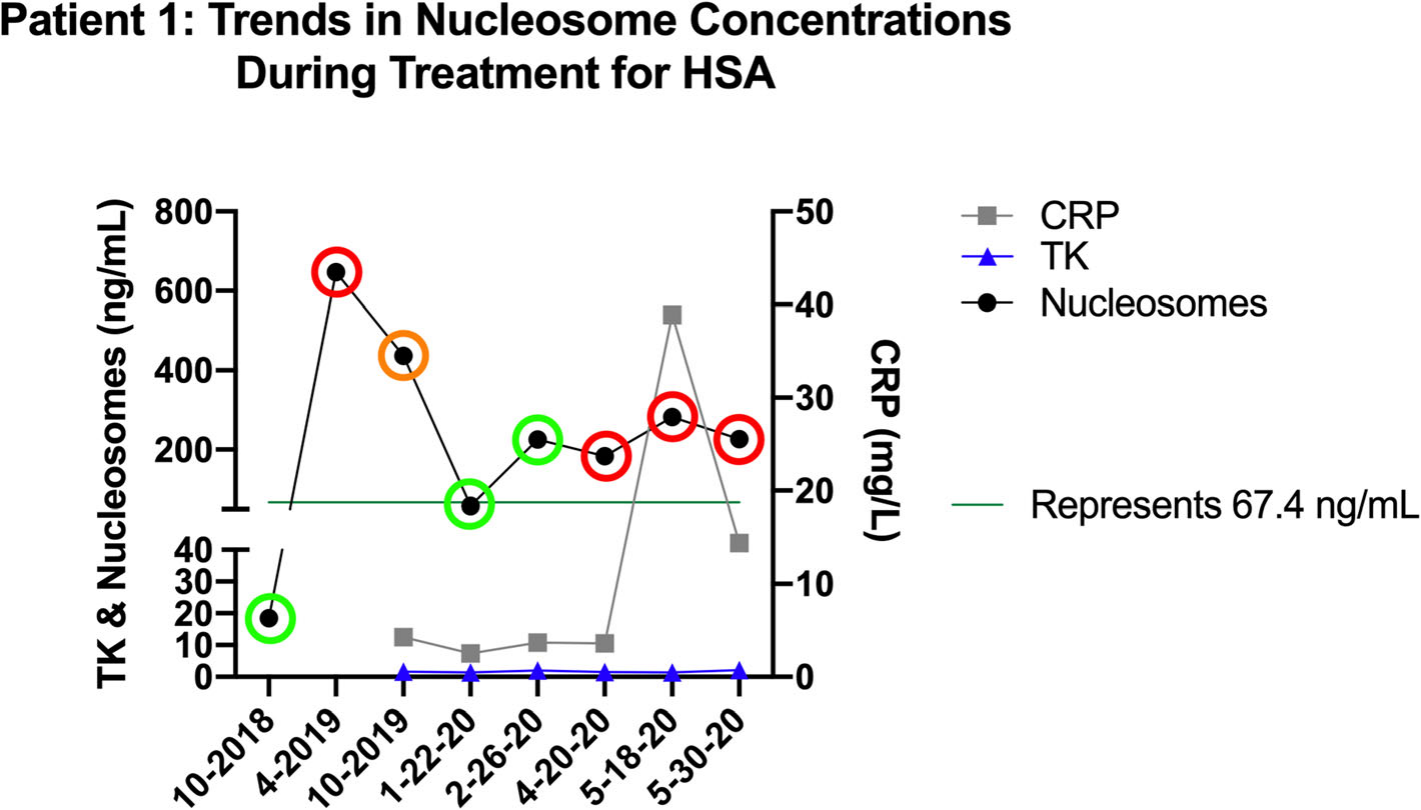
Longitudinal assessment of nucleosome concentrations in a patient with stage III HSA throughout the course of treatment. The green circles represent clinical remission, red circles represent progressive disease and the orange circle represent a partial clinical response. All assessments were performed by a board-certified veterinary medical oncologist. All imaging was interpreted by a board-certified veterinary radiologist

Serial plasma samples from one dog with recurrent stage III HSA were evaluated throughout the course of treatment (Fig. 5). This patient was a 12-year-old MN Catahoula hog dog that had previously been diagnosed and treated for subcutaneous HSA in 2017. Through routine monitoring (whole body CT scan every 2–3 months) he was found to have progressive disease in April of 2019 with multiple pulmonary and intramuscular lesions as well as an intraseptal intracardiac lesion. Cytology on an intramuscular metastatic lesion was consistent with HSA and this was later confirmed at necropsy. This patient underwent several therapeutic interventions including stereotactic radio-body therapy, whole lung radiation, chemotherapy (doxorubicin, dacarbazine, chlorambucil and vinblastine), alpha- and beta-adrenergic blockade and immunomodulatory therapy over the course of the next year. He was in clinical remission in January of 2020 until April of 2020 where progressive disease was noted once more on imaging. Interestingly, his plasma nucleosome concentrations began to increase in February, 2 months before progression was noted on imaging and remained high even though additional therapies were attempted. Unfortunately, the patient failed to respond to the additional therapies and was euthanized secondary to complications of central nervous system metastases at the end of May 2020. Necropsy confirmed widespread metastatic disease. Plasma samples were collected before progression was noted in October of 2018 and throughout treatment. These samples were processed according to predetermined optimal criteria and frozen at -80 C [16]. The samples were batched and all run together after the patient’s death in conjunction with C reactive protein (CRP) and thymidine kinase (TK) assays. Interestingly, plasma nucleosome concentrations reflect the clinical course of disease more accurately than CRP or TK, at least in this one patient. There is a spike in CRP noted with a small increase in nucleosome concentrations in May of 2020 for this patient which coincided with an episode of severe gastroenteritis that required ICU hospitalization for 2 days.

## Discussion

HSA is a common mesenchymal malignancy often affecting larger breed dogs. This disease is characterized by a primary cavitated lesion in the viscera (spleen, heart, liver), muscular or subcutaneous tissue with a high propensity to spread early and often to other locations in the body such as the lungs, lymph nodes and other viscera [20, 21]. Because this is a tumor of blood vessels, a sudden onset of hypovolemic shock due to acute spontaneous bleeding maybe the first clinical sign observed by the pet owner [24]. Non-invasive diagnostics may be highly suggestive of HSA; however, it is very difficult to collect cytologic or biopsy samples of these tumors without a surgical approach in many cases due to the fragility of the tumor and the extraordinary amount of blood flow the tumors possess [21, 25]. For this reason, new tests that can help direct client decision making without increasing the risk of acute hemorrhage are needed.

Though nucleosomes have DNA wrapped around them, they stand apart from other forms of circulating DNA through their pathophysiology. Nucleosomes play an active role in sepsis as part of neutrophil extracellular traps (NETs) which serve to remove extracellular pathogens from the blood [26]. In cancer, they are suspected to play a role in metastasis and injections of nucleosomes into mice with xenografts led to increased metastatic lesions and new tumor manifestations [27]. They have also been shown to enhance endothelial cell expression of interleukin 8 leading to neoangiogenesis in tumor tissue and ultimately tumor progression [28]. Finally, they have also been shown to play a role in tumor cell immune evasion by inhibiting natural killer cell-mediated tumor lysis [29].

Though not specific for HSA, elevated nucleosome levels in the plasma of patients with HSA may provide additional information that can be useful to the client and veterinarian alike. In this cohort of 77 dogs, 80.5 % of them (62 of 77) had mean plasma nucleosome concentrations above the 67.5 ng/mL designated cut off which is higher than the nucleosome levels of all 134 healthy dogs included in this study. A previously published manuscript evaluating the nucleosome compartment of healthy dogs determined that factors such as fasting, processing time, sample type and storage conditions can alter nucleosome concentrations in the plasma samples of healthy dogs [16]. For this reason, special care was taken in the selection and handling of these samples to ensure that all samples were handled the same way. When properly prepared, the plasma samples collected from normal dogs showed very little variability, similarly to what is seen in humans^7^.

The cohorts of both healthy dogs and those diagnosed with HSA were fairly diverse representing a variety of ages, breeds, body sizes and genders. However, nucleosome levels were unaffected by these factors (see supplement data). Only a diagnosis of HSA was able to differentiate between the two groups. Furthermore, the mean and median plasma nucleosome concentrations in the healthy dogs were surprisingly consistent across a variety of ages, body sizes and genders (supplement data) with SEMs ≤ 5 ng/mL in all categories evaluated except body weight where dogs weighing more than 45 kg had an SEM of 14.7 ng/mL. These data demonstrate that the nucleosome compartment is consistently low in healthy dogs across the board.

In this study nucleosome concentrations varied by location; however, these results should be viewed with caution for all locations except splenic HSA due to the low numbers of cases in the other groups. Interestingly, 100 % (5/5) of the primary bone HSA cases were elevated. A study evaluating 48 cases of osteosarcoma (OSA) by this group demonstrated elevated nucleosome concentrations in only 17/48 cases (35 %) with the highest levels in those cases with metastasis (data not shown). More cases of primary osseous HSA are needed; however, this assay may be able to differentiate HSA from OSA when it is localized to the bone.

Nucleosome concentrations are predictive of stage in humans in gastrointestinal cancer, while there is no correlation to stage for other cancer types [30]. In humans with lung and breast cancer, even the early stages of disease were found to have elevated serum nucleosome concentrations, similar to what was found here in dogs with HSA [31]. In the present study, elevated nucleosome concentrations were detected in 67 % of dogs with stage I disease, 76 % of dogs with stage II disease and 90 % of dogs with stage III disease. This ability to detect early stage disease can be particularly useful as a screening test for older dogs by possibly detecting HSA before there is an acute bleeding episode.

Like most types of cfDNA, nucleosomes are cleared from the plasma quickly, usually within an hour [3, 4]. In healthy individuals’ nucleosomes are often cleared within minutes through endonuclease degradation, immunocomplexing with anti-nucleosome antibodies, phagocytosis and lysosomal degradation, hepatic metabolism or direct excretion through the kidneys [4]. However, in diseased patients, nucleosome elimination may be delayed when they bind to acute phase proteins [4]. Though cancer is not the only cause of elevated nucleosome concentrations in humans or dogs, the short half-life of this type of cfDNA can provide a real time window into the amount of apoptosis or cell turn over in that patient at any given time. Elevations in plasma nucleosomes have been used in humans for monitoring response to therapy or recovery in a variety of diseases such as autoimmune disease, cancer, sepsis, viral infections and trauma [3, 30, 32–34]. For this reason, plasma nucleosome concentrations may be a reliable and effective way to screen otherwise healthy canine patients at risk for HSA. Caution should be used; however, when evaluating plasma nucleosome concentrations of clinically ill dogs as nucleosomes are not specific for HSA or other common cancers.

Nucleosome concentrations have been used to monitor treatment response in rodents with HeLa xenografts as well as humans with cervical cancer and were found to reliably predict responders in both groups [35]. Circulating nucleosome concentrations went down significantly after one cycle of platinum-based chemotherapy in the women with cervical cancer who had measurable responses. In patients with acute myeloid leukemia undergoing induction chemotherapy, nucleosome concentrations were an early predictor of treatment responders [36]. Plasma nucleosome concentrations also correlated with response to therapy in patients with non-small cell lung cancer [37, 38]. Here, we present a single case as a proof of concept that plasma nucleosome concentrations may serve as a surrogate for therapeutic response in patients with HSA. Though many more data points are needed before the utility of this test can be applied as a treatment monitoring tool for dogs with HSA, it is promising that nucleosome concentrations in dogs may mirror that which is seen in humans with a variety of cancers.

Other proteins, such as C-reactive protein (CRP) and Thymidine kinase have also been used to diagnose and monitor disease status in dogs with HSA. CRP is an acute phase protein that has been previously reported as a useful tool for evaluating remission status in canines with lymphoma and HSA [39–41]. Thymidine kinase is an enzyme involved in pyrimidine synthesis and increases in extracellular TK activity could indicate the overall degree of DNA synthesis and dying cells. TK has also been reported as a useful marker of remission status in canine lymphoma patients [39]. We evaluated both CRP and TK along with nucleosome concentrations in this patient as a proof of concept and found that CRP and TK values did not significantly change during the course of therapy and were not correlated with the clinical response in this one patient. Additional cases are needed to determine how effective nucleosomes concentrations are for disease monitoring when compared to CRP and TK.

## Conclusions

Plasma nucleosome concentrations are an effective way to differentiate dogs with HSA from their healthy counterparts. Plasma nucleosome concentrations are fairly consistent in healthy dogs regardless of age, gender or body size (supplement data) and elevated nucleosome concentrations were seen even in early stage disease in over 80 % of the patients evaluated. Common signalment characteristics did not affect nucleosome concentrations in dogs with HSA when compared to healthy dogs. Measuring circulating nucleosome levels provides significant promise as a non-invasive tool for detecting HSA while limiting the likelihood for complications that are common with vascular tumors.

## Materials and methods

### Animal samples

All animal studies were approved by the Texas A&M University Institutional Animal Care and Use Committee (AUP# 2019 − 0211 and AUP# 2017 − 0350). Additional samples were purchased from the National Cancer Institute Division of Cancer Treatment and Diagnosis (NCI-DCTD) biospecimen repository. A total of 134 healthy dog volunteers were recruited from TAMU-SAC staff and client pets. A total of 77 samples from dogs with confirmed HSA were also recruited or purchased for this study. Informed consent was obtained from all pet owners before participation in the study.

### Sample collection and processing

All blood samples were collected from a peripheral or jugular vein using standard blood collection techniques. All dogs were fasted for a minimum of 6 h before samples were collected. A total of 3–5 mL (depending on the size of the dogs) were placed in EDTA primed (lavender top) tubes (Becton, Dickinson and Company, Franklin Lakes, NJ). Samples were centrifuged within one hour of collection at room temperature at 3000xg for 10 min [16]. Plasma was then immediately removed without disrupting the buffy coat layer, placed in pre-labeled cryovials and frozen at -80 °C to run in batches. All samples were run in duplicate.

All samples were tested using the Nu.Q™ H3.1 assay (Belgian Volition, SRL, Isnes, Belgium). This enzyme-linked immunosorbent assay (ELISA) contains a capture antibody directed at histone 3.1 and a nucleosome specific detection antibody [42]. Frozen samples were thawed and allowed to come to room temperature for at least 30 min prior to analysis. Assays were performed according to the manufacturer’s protocol. Briefly, a standard curve was generated using the positive control stock (recombinant H3.1 nucleosomes) provided. Samples were vortexed and centrifuged at 11,000 x g for 3 min at 4 °C. HSA samples were diluted 3-fold to ensure that they could be measured within the detection limits of the assay. The nucleosomes were bound to the capture antibody and the plates were washed 3 times using 1x wash buffer. Twenty microliters of each diluted sample were pipetted in duplicate into wells on the 96 well plates. Next, 90µL of the assay buffer was added to each well. The plate was covered with sealing film and incubated on an orbital shaker for 2.5 h at 700 rpm. Plates were then emptied and washed 3 times using 1x washing buffer. Next, 100 uL of the detection antibody was added to each well, the plate was resealed and incubated for 1.5 h on the orbital shaker. The plates were then washed as described above. Streptavidin HRP conjugate was incubated for 30 min in each well and washed before applying the colorimetric substrate solution and incubating the plates in the dark for 20 min. A stop solution was added to the wells and the plates were read on a plate reader at 450 nm (BioTek Synergy H1 plate reader, BioTek Instruments, Winooski, VT). The standard curve was linearized and fitted to a 4-parameter logistic curve using statistical software (Graphpad Software, version 8, San Diego, CA).

One patient was followed longitudinally through his treatment protocol with serial sampling. C-reactive protein (CRP) and thymidine kinase (TK) were also measured on the samples collected at these appointments. The CRP assays were performed by the Texas A&M University Gastrointestinal Laboratory and the TK assay was performed using the Canine Thymidine Kinase 1 soluble ELISA assay (My Biosource Inc, San Diego, CA) as long as sufficient sample was available to do so.

#### CRP Assays

For the longitudinally followed patient, samples were submitted to the Texas A&M University Gastrointestinal Laboratory for c-reactive protein (CRP) assays if sufficient sample quantity was present. If sample quantity was not sufficient for both nucleosome and CRP analysis, nucleosome assays were given priority.

#### Thymidine kinase assays

The Canine Thymidine Kinase 1 soluble ELISA assay (MyBiosource Inc, San Diego, CA) was used to evaluate TK1 levels in the dog followed longitudinally. The assay was performed according to the manufacturer’s protocol. Briefly, 40 µl of sample was added to wells followed by 10 µl anti-TK1 antibody. Then 50 µl streptavidin-HRP was added to each well except the blank control well. The plate was mixed well, covered with sealer and incubated for 60 min at 37 °C. The plate was then washed 5 times with wash buffer and the wells were soaked with at least 0.35 ml wash buffer for 30 s to 1 min for each wash. Next 50 µl of substrate solution A was added to each well followed by 50 µl of substrate solution B to each well. The plate was covered with a fresh sealer for 10 min at 37 °C in the dark. Finally, 50 µl of Stop Solution was added to each well. Plates were read at an absorbance of 450nm (BioTek Synergy H1 plate reader, BioTek Instruments, Winooski, VT) within 10 min of stop solution being added. The standard curve was linearized and fitted to a 5-parameter logistic curve using statistical software (Graphpad Software, version 8, San Diego, CA).

#### Intra and intergroup comparisons

HSA samples were separated out by stage, age, weight and gender and compared used the statistical methods below. Stage I HSA is characterized by a solitary lesion that is < 5 cm in diameter, no evidence of hemorrhage and no evidence of metastasis (locoregional or distant) [43]. Stage II HSA is characterized by a lesion that is either 5 cm or greater or one that has ruptured with or without locoregional metastasis and no distant metastasis [43]. These tumors may also be invading into the subcutaneous tissues. Stage III HSA is characterized by highly invasive tumors with or without distant metastasis or tumors of any size with distant metastasis [43]. Age was divided into 4 groups: <3 years, 4–7 years, 8–11 years and > 12 years. Gender was also divided into 4 groups: spayed females, intact females, neutered males and intact males. Finally weight was also divided into 4 groups: <15 kg, 15–30 kg, 30–45 kg and > 45 kg.

### Statistical analysis

Descriptive statistics for the patient populations were performed using Microsoft Excel for Mac (v. 16.16.27, 2016). For data sets containing only two cohorts, such as the healthy controls versus all HSA cases, a Wilcoxon rank sum test was used to compare the medians of the data sets. For data sets where multiple conditions were compared, such as disease stage, a two-way ANOVA for repeat measures with a Tukey’s multiple comparisons test was performed. This part of the analysis was performed using GraphPad Prism version 8.0.0 for Macintosh, GraphPad Software, San Diego, California USA, www.graphpad.com. Wilcoxon rank sum tests, ROC curves and specificity/sensitivity calculations were performed using R version 3.4.3 and the pROC package [44, 45].

## Supplementary Information

**Additional file 1.**

## Data Availability

All relevant data are within the paper.

## Abbreviations

AUC: Area under the curve
CA125: Cancer antigen 125
CEA: Carcinoembryonic antigen
cfDNA: Cell free deoxyribose nucleic acid
CRP: C-reactive protein
EDTA: Ethylenediaminetetraacetic acid
ELISA: Enzyme linked immunosorbent assay
HSA: Hemangiosarcoma
ICU: Intensive care unit
LB: Liquid biopsy
NCI-DCTD: National Cancer Institute Division of Cancer Treatment and Diagnosis
NETs: Neutrophil extracellular traps
PSA: Prostate specific antigen
ROC: Receiver operator characteristic
SEM: Standard error of mean
TK: Thymidine kinase
