# Supplementary material for: Characterizing circulating nucleosomes in the plasma of dogs with hemangiosarcoma
Source: BMC Vet Res. 2021 Jun 29;17:231. doi: 10.1186/s12917-021-02934-6 (PMC8243913; doi:10.1186/s12917-021-02934-6)
Supplement: Supplementary file 1 — Additional file 1. [file 12917_2021_2934_MOESM1_ESM.docx]

**Supplemental Data**

Due to the lack of published information regarding the plasma nucleosome compartment in healthy dogs, additional data points were compared within this population of 134 healthy dogs. These dogs were separated into similar groups based on signalment, similar to what was performed in the dogs with HSA (tables 1-3) in the main text, in an effort to better define whether certain factors such as weight, age or gender affects the nucleosome compartment in healthy dogs. For these comparisons, all categories were compared to each other and the lowest p value from each comparison is listed in the tables.

For the healthy dogs there were 11 dogs less than 4 years old (8.2%), 40.2% (54/134) were between 4-7 years of age, 38% (51/134) were 8-11 years of age and 18 (13.4%) were 12 years or older. This group, on average, was younger than the cohort of dogs with hemangiosarcoma, however, there was no statistical difference in the median plasma nucleosome concentrations for any of the age groups.

Supplemental Table 1: Nucleosome concentrations in healthy dogs separated by age.

|  | up to 3 years | 4 years up to 7 years | 8 years up to 11 years | Greater than 12 years |
| --- | --- | --- | --- | --- |
| Number of cases | 37 | 53 | 29 | 8 |
| Median | 32.0 | 28.9 | 29.1 | 28.3 |
| Mean | 34.0 | 32.0 | 29.2 | 28.3 |
| SEM | 1.6 | 2.0 | 2.4 | 3.4 |
| P value | 0.248 | 0.659 | 0.310 | 0.455 |

When comparing gender in the cohort of healthy dogs, the same trend was also evident. There was no significant difference between the groups, though two of the groups (intact females and intact males) were too small for meaningful comparisons. When comparing the two largest groups (spayed females and neutered males) the p value was 0.29.

Supplemental Table 2: Nucleosome concentrations in healthy dogs separated by gender.

|  | Female Spayed | Female Intact | Male Neutered | Male Intact |
| --- | --- | --- | --- | --- |
| Number of cases | 61 | 4 | 65 | 3 |
| Median | 29.5 | 38.3 | 31.6 | 19.5 |
| Mean | 30.6 | 33.9 | 33.6 | 20.8 |
| SEM | 1.6 | 0.6 | 1.6 | 2.3 |
| P value | >0.35 | >0.35 | >0.78 | >0.99 |

Median plasma nucleosome concentrations also were similar in the healthy cohort when separated out by weight. The healthy cohort does contain more small breed dogs than the hemangiosarcoma cohort as hemangiosarcoma tends to affect larger breed dogs. There were 18 dogs (21.1%) that weighed <15 kg, 27% (23/85) of the dogs weighed 15-30 kg, 16.4% (14/85) dogs weighed 30-45 kg and only 3 of the dogs (3.5%) weighed more than 45 kg. However, there was very little difference in the mean nucleosome concentrations between any of the groups.

Supplemental Table 3: Nucleosome concentrations in healthy dogs separated by weight.

|  | up to 15 kg | above 15  up to 30 kg | above 30  up to 45 kg | above 45 kg |
| --- | --- | --- | --- | --- |
| Number of cases | 19 | 47 | 18 | 3 |
| Median | 24.4 | 30.2 | 29.4 | 25.2 |
| Mean | 26.9 | 32.3 | 30.5 | 33.9 |
| SEM | 2.6 | 2.2 | 2.4 | 14.7 |
| P value | 0.374 | 0.2194 | 0.2194 | 0.5931 |
